# Using a learning community model for virtual medical student support during the COVID19 pandemic

**DOI:** 10.5116/ijme.60e2.c777

**Published:** 2021-07-27

**Authors:** Emily Frosch, Mitchell Goldstein

**Affiliations:** 1 Department of Psychiatry and Behavioral Sciences, Division of Child and Adolescent Psychiatry, Johns Hopkins Universi-ty School of Medicine, Baltimore MD, USA; 2 Department of Pediatrics Division of Pediatric Emergency Medicine, Johns Hopkins University School of Medicine, Baltimore MD, USA

**Keywords:** Learning community model, virtual medical student, support, COVID19, pandemic

## Introduction

The COVID-19 pandemic has impacted medical education in an unprecedented way across the globe. On March 17, 2020 in the United States, The Association of American Medical Colleges (AAMC) made the recommendation to remove all medical students from clinical training. At the same time, medical schools shifted preclinical training from the classroom to distance learning.  Not only did these changes completely disrupt the medical school curriculum, but they also completely transformed the learning environment. Suddenly the familiar, in-person relationships so vital to maturation and identity formation had been seemingly severed. This move, prioritizing student safety, deposited students and educators alike into uncharted territories.

Medical school is an inherently complex period with steep learning curves, a dramatic shift in identity formation from a focus on self to an assumption of increasing responsibility for the lives and well-being of others, long hours and intense interpersonal experiences. The explosive growth of Learning Communities (LC) across medical school campuses in many countries in the last decade highlights the growing value placed on relational connections as the key foundation for making sense of this multilayered experience.^1^ Learning communities are organized in various ways across schools yet share a focus on longitudinal connections for the purpose of supporting student professional development. Many LCs facilitate the sense of connection through creating smaller units or groups such as “houses” or “colleges.” This structure can make academic and career planning, milestone celebrations, advising around transitions, and supporting well-being more personal. LC’s thus can offer community structures that create connections between peers and faculty.^2^ Gathering in advisor-advisee dyads or small groups, often in dedicated spaces, can help students make sense of the many personal and professional transformative experiences embedded in medical school, which is crucial in the development of their professional identity. During a pandemic, however, new challenges and uncertainties arise for students, faculty, and institutions as the once familiar advising landscape can appear very different. The principles of relationally-focused advising embedded in Learning Communities offer a framework for developing an updated road map in a virtual environment.

As Coronavirus took hold, both medical school administrators and students were forced to make rapid decisions based on limited and rapidly evolving information. Students had to decide whether to stay on campus or go home. This decision had many others embedded within it such as 1) travel considerations—different states imposed different rules, international students faced travel restrictions that appeared overnight, 2) family considerations-- what are the risks to others at home including elderly relatives, those with illnesses or otherwise vulnerable. For some, home may not be a refuge during these uncertain times but a return to environments not conducive to learning or even potentially toxic, 3) personal well-being – exposure risks, physical and mental health vulnerabilities that may be exacerbated during these uncertain times, familial/partner loss of employment, family illness or death, and loss of social networks posed by the virus’ spread may all add to the distress, and 4) existential crises—should I be contributing somehow? What will others think if I don’t? Will this impact my graduation timeline? At the same time, faculty advisors faced similar acute stressors related to the pandemic. For some, their own professional roles were thrown into chaos. Research faculty suddenly were shut off from their work, teachers needed to rework courses overnight to proceed in a virtual environment, and clinicians may have discovered they were re-deployed to unfamiliar care settings. Work schedules became less predictable, actual work changed for many and concerns for personal and patient safety came with being in front-line work. Some worried about job certainty within institutions for whom the economic challenges to the health care system seem insurmountable or for others in their household whose work may have been differentially impacted by the shutdowns. In addition, faculty parents suddenly found themselves in the role of teachers or stay at home parents with their children while still working and trying to figure out a whole new approach to work-life balance.  All of this made navigating advising roles even more challenging.

Institutionally, leaders at various levels needed to coordinate and make decisions that would have large ripple effects with limited information. Meetings increased, the discussion was held, decisions made, sometimes changed within hours or days as new information was learned. Cross-institution collaboration increased even though state lines accentuated differences. Previously available senior leaders were now focused on the acute steps that needed to be implemented to save lives and be prepared.

Worries about exposure and illness, schedules and career momentum, physical, emotional, and financial health are real for all involved to varying degrees. Tolerating uncertainty and ambiguity, a common component of being a physician, is magnified by the pandemic with limited and changing knowledge, experience, and guidelines in the face of palpable risks to all stakeholders. George Everly, PhD, has described the risk for psychological toxicity post event may be at least in part driven by the interaction between lethality, chronicity, and ambiguity in any given situation.^3^ The current pandemic could easily be rated high in each of those domains.

Despite these myriad challenges to faculty and learners alike, the scaffold set up by learning communities pre-pandemic can help nourish and sustain advising in this new landscape. LCs can offer a grounding for faculty though their authentic connections with colleagues. That, in turn, can facilitate the faculty in feeling ready and able to offer the needed support and guidance to students. Both advisors and advisees alike appreciate the constants in their professional and personal work, and the advisee-advisor dyad can serve as a tie to normalcy for both.  As an authentic, longitudinal relationship, it can help serve as a mediator for some of these stressors. In the current setting of social disconnection, loss, and disruption, it may be helpful to look to models more typically considered post-traumatic events. The primary goals of Psychological First Aid (PFA), a model of post-event intervention widely accepted by the World Health Organization and other international bodies, are to mitigate acute distress and instill hope and foster positive, future-focused orientation. While not a framework that is typically used in programs designed to support medical students, PFA may offer a useful framework to consider given the profound impact of Covid19.  The key elements that it highlights as the means to reach these goals include active listening to ensure empathy which leads to understanding and the building of trust.^4^ These are not only the building blocks of PFA. They are also the essential elements of relationally anchored advising in a learning community.^5^ Using a PFA lens may be useful in framing ways to support students in the new virtual environment.

While the onset of this pandemic has been acute and abrupt, the offset is likely to be much slower. The initial surge of supporting front-line staff and rallying behind mask making and social distancing practices will yield to reflection on the loss, the grief, and the changes in how we all live and work—both as students and physicians but also as individuals. Given that, and the likelihood that this virus will pose a persistent problem, it is crucial that we reframe our advising practices. We must consider how to approach medical student advising in ways that build on our past models yet embrace the acute change as well as the ongoing stressors and inevitable persistence of uncertainty while also working together to bolster resilience and identify sustainable adaptations.

### So what can we do?

Although in-person advising is suspended, one-on-one advising can occur with face-to-face contact with a portal like Zoom, phone calls or texting.  Advisors need to check in with individual advisees and make impromptu advising sessions even more available during this time.  Advisors should assume that each advisee needs even more frequent touchpoints.  In addition, group advising may become even more important. Not all students and faculty have access to virtual portals. However, there is a range of options for connecting virtually, including cell phones, group chats, and other more accessible means that can make staying connected easier.

The content of individual advising should follow the same architecture of pre-COVID 19 sessions but with changes in strategy. The main domains to consider include personal check-ins, curricular discussions, advising around specialty choice and the match, discussions on research, community, and extracurricular identities. Personal check-ins need to center on advisees personal needs. These include personal safety, physical, emotional, and economic wellness, relationships as well as screening for mental health concerns including grief, loss, anxiety, depression, and substance misuse.

Students’ fledgling professional identities and their sense of who they are in the world may have been suddenly transformed as they return to their families to resume studies in their childhood bedrooms, or perhaps in homes that are absent of the daily reminders of who they are hoping to become.  What an opportune time to reflect on the two worlds in which they are suddenly immersed at once. What strengths can they draw from each to inform their emerging sense of self and physician-hood? Additionally, there are likely disparities that will arise among students in returning to their homes as some may feel less supported, more vulnerable, or otherwise disadvantaged, providing a need for exploration and support from their advisor. Some students may not return home at all—perhaps for positive reasons, perhaps for worrisome ones. Understanding the narrative each student creates about this period can be helpful to see how they are incorporating it into their personal story, sense of self-efficacy, and future planning.

The cohesive dynamics of learning community small groups can serve as important social outlets and offer vital peer support during these periods of isolation.  Dialog with others in the same situation or facing similar worries and concerns can be grounding in difficult times.^6^ The vertical and peer relationships naturally facilitated by LCs may thus be a familiar source of comfort and may play a role in normalizing responses and creating connections through sharing.

Curricular discussions would include adapting learning styles for distance learning, how to navigate online elective opportunities, and research possibilities. Advising around the match would include the timing of licensing exams with the uncertainty of test site availability, opportunities for clinical experiences that would inform career trajectory when clinical opportunities are not available or altered. Community and extracurricular discussions would include service to the community during a pandemic and putting personal health at risk, balancing research opportunities, and wellness activities during the stay at home or safe at home periods. Just as not being able to rotate on clinical services brings stressors, so will returning to clinical rotations. Creating space to air those concerns, providing accurate information that can help assuage worries, and supporting the shared holding of the unknown can be important steps as students re-enter clinical services.

Another area in which advisors in LCs can support students is in giving them permission to be distressed. Rapid disruptions in living and learning can be hard to assimilate, and students may welcome permission to struggle, permission to worry, permission to feel less motivated or fatigued by the technology-only interactions that are happening. Many students are highly conscientious “planners” who may find this degree of uncertainty particularly uncomfortable or unfamiliar. Helping to normalize such feelings can be a powerful aid as they try to find their footing. The active listening and empathic understanding that LC faculty bring to their connections with students can facilitate processing these emotions in a constructive way. Faculty also have an opportunity to model coping with uncertainty, doubt, and fear as a person and as a physician. The bi-directional nature of relationally anchored advising creates a unique opportunity to share what we as faculty are trying out in our own personal-professional lives

LC’s can use the pre-existing collegial and collaborative faculty structure not only as a scaffold for faculty peer support but also as channels for information sharing. Faculty need to stay alert to the changing timelines, regulations, and decisions from their own institution and the many external bodies that deeply impact students’ decisions and timelines. In the United States, organizations such as AAMC, LCME, ERAS, NRMP, and NBME are actively updating policies and guidelines.  How this information is disseminated may vary from school to school, but it can be helpful for advisors to familiarize themselves with the key organizations that directly impact student options. Town halls are one method for sharing information, and other methods may serve some school communities better. Community gatherings, even virtual ones, can be helpful in modelling how to manage uncertainty and the associated vulnerability that comes with not knowing the role of health professionals and leaders.  Faculty advisors may be important advocates in facilitating such discourse at their own institution.  Student leaders are important stakeholders and may assist in coordinating these types of activities. In addition, such forums offer moments to demonstrate the cooperative engagement between the various arms of the institution’s educational world—student affairs curriculum offices, technology offices, etc., along with the LC leaders.

Although the COVID-19 pandemic has brought with it more questions than answers and a total upheaval to how we live and work, the stable foundation that learning communities bring to a medical school campus has only become clearer. The learning community model of relationally anchored advising that has greatly expanded throughout medical schools across the world—Mexico, Canada, Lebanon, and other countries, can create a scaffold for providing support, guidance, and genuine connection during the peculiar and uncertain times in which we currently find ourselves. Administrators, faculty and students alike can harness the strengths of this model to not just get through but to thrive during these unprecedented times. Many settings may not have an LC structure in place or have access to some of the resources described. Despite that, maximizing connection through whatever means possible and being attuned to the various ways this pandemic may be impacting students (and staff and faculty) may help mitigate isolation and distress.

### Conflict of Interest

The authors declare that they have no conflict of interest.
